# Aerobic exercise program with or without motor complexity as an add-on to the pharmacological treatment of depression – study protocol for a randomized controlled trial

**DOI:** 10.1186/s13063-018-2906-y

**Published:** 2018-10-10

**Authors:** Lucas Melo Neves, Carla Silva-Batista, Raquel Marquesini, Telma Fátima da Cunha, Elisa Dimateo, Luciana Nascimento, Acácio Moreira-Neto, Angelo Corrêa de Lima Miliatto, Sheila das Chagas Mendes, Flavia Saad, Jamile Sanches Codogno, Renato Hoffmann Nunes, Raphael Mendes Ritti-Dias, Valeria Juday, Beny Lafer, Carlos Ugrinowitsch

**Affiliations:** 1 School of Physical Education and Sport, University of São Paulo (EEFE-USP), São Paulo, Brazil; 2 Universidade Paulista (UNIP), São Paulo, Brazil; 3 Federal University of São Paulo (UNIFESP), São Paulo, Brazil; 4 São State University (UNESP), Presidente Prudente, Brazil; 5 Medical Radiologist of DASA, São Paulo, Brazil; 6 Faculty of Medical Sciences Santa Casa de São Paulo, São Paulo, Brazil; 7 Universidade Nove de Julho, São Paulo, Brazil; 8 Anhembi Morumbi University (UAM), São Paulo, Brazil; 9 Department of Psyquiatric, University of São Paulo (IPq-USP), São Paulo, Brazil

**Keywords:** Depression, Cognitive function, Neuroplasticity, Brain volume, Cardiovascular, Metabolic syndrome, Coordinative exercise, Clinical trial, Physical activity level

## Abstract

**Background:**

Patients with major depression disorder presents increased rates of cognitive decline, reduced hippocampal volume, poor sleep quality, hypertension, obesity, suicidal ideation and behavior, and decreased functionality. Although continuous aerobic exercise (CAE) improves some of the aforementioned symptoms, comorbidities, and conditions, recent studies have suggested that performing aerobic exercise with motor complexity (AEMC) may be more beneficial for cognitive decline, hippocampal volume, and functionality. Therefore, this randomized controlled trial will compare the effects of CAE and AEMC on depression score, cognitive function, hippocampal volume, brain-derived neurotrophic factor expression, sleep parameters, cardiovascular risk parameters, suicidal behavior, functionality, and treatment costs in patients with depression.

**Methods/design:**

Seventy-five medicated patients with depression will be recruited from a Basic Healthcare Unit to participate in this prospective, parallel group, single blinded, superiority, randomized controlled trial. Patients with depression according to DSM-V criteria will be balanced and randomly assigned (based on depression scores and number of depressive episodes) to a non-exercising control (C), CAE, and AEMC groups. The CAE and AEMC groups will exercise for 60 min, twice a week for 24 weeks (on non-consecutive days). Exercise intensity will be maintained between 12 and 14 points of the rating of perceived exertion scale (~ 70–80% of the maximum heart rate). The CAE group will perform a continuous aerobic exercise while the AEMC group will perform exercises with progressively increased motor complexity. Blinded raters will assess patients before and after the intervention period. The primary outcome measure will be the change in depression score measured by the Montgomery-Asberg Depression Rating Scale. Secondary outcomes will include measures of cognitive function, hippocampal volume, brain-derived neurotrophic factor expression, sleep parameters, cardiovascular risk parameters, suicidal behavior, functionality, and treatment costs.

**Discussion:**

This study was selected in the call of public policy programs for the Brazilian Unified National Health System – “PPSUS 2015”. To our knowledge, this is the first pragmatic trial to test the effect of adding AEMC to the pharmacological treatment of patients with depression and to evaluate the possible reductions in depression symptoms and healthcare costs.

**Trial registration:**

Brazilian Clinical Trials Registry (ReBec) - RBR-9zgxzd - Registered on 4 Jan. 2017.

**Electronic supplementary material:**

The online version of this article (10.1186/s13063-018-2906-y) contains supplementary material, which is available to authorized users.

## Background

Major depressive disorder (MDD) is a mood disorder characterized by several behavioral and emotional symptoms, such as depressed mood, irritability, anhedonia, psychomotor agitation or lethargy, insomnia or hypersomnia, decreased appetite, fatigue, and suicidal ideation and behavior [1]. This disorder is a global health burden in which the cost of the traditional pharmacological treatment will reach approximately US$ 91 billion in 2030 [2]. In addition to direct costs, MDD usually occurs in association with several comorbidities.

Epidemiological and review studies have reported a relation between depression and decreases in cognitive function [3–6], brain volume, and expression of brain-derived neurotrophic factor (BDNF) [7]. In addition, the odds ratio of sleep disturbances (1.42 to 3.23), short sleep duration (1.41 to 2.53) [8], metabolic syndrome (1.58), and obesity (1.27) [9, 10] is significantly higher for MDD patients than for the general population. Furthermore, 9 out of 10 suicides are related to psychiatric illness [11, 12], with depression being observed in two-thirds of them [13].

As a large portion of patients with depression do not properly respond to drug treatment (30% response without remission, 20% partial remission and 50% remission) [14], alternative interventions should be added to standard drug treatment aiming to improve the health status of patients. Non-pharmacological synergic interventions, such as physical exercise, have been deemed effective to mitigate the burden of depression and associated comorbidities [15, 16]. Accordingly, physical exercise has been widely recommended as an effective non-pharmacological intervention for depression, cognitive function, hypertension, obesity, and type 2 diabetes [17, 18]. The effectiveness of physical exercise to decrease the burden associated with these conditions has been confirmed by several systematic reviews and meta-analyses [16, 19–22]. For example, Cooney et al. [16] (35 studies, 1.353 subjects) and Wegner et al. [19] (32 studies, 48,207 subjects) reported that aerobic exercise can reduce depression symptoms (effect sizes of 0.62 and 0.56, respectively). Smith et al. [20] (31 studies, 2.049 patients with depression) demonstrated that physical exercise produces small improvements (in elderly people) in indices of cognitive function, such as attention and processing speed (effect size 0.16), executive function (effect size 0.12), and memory (effect size 0.12). Furthermore, Cornelissen and Smart [21] found a reduction in systolic blood pressure of healthy adults after aerobic exercise (93 studies, 5223 subjects).

Even though continuous aerobic exercise (CAE) seems to produce beneficial effects on depression and associated comorbidities, recent studies have shown that physical exercise with high motor complexity may produce additional benefits to overall health status. Specifically, exercise with high motor complexity may be particularly beneficial for patients with depression as it requires high levels of attention, memory, and motor difficulty, producing greater cortical activation than exercises with low motor complexity (e.g., CAE) [23, 24]. Accordingly, high cortical activity (i.e., due to the integration of perceptual, motor, and cognitive information) has the potential to produce greater neuroplasticity [25] (i.e., changes in brain structure and function) than conditions requiring low cortical activity (low motor complexity). Recent studies from our group demonstrated that 3 months of an exercise program with high motor complexity produced greater cognitive improvement in patients with Parkinson’s disease than exercises with low motor complexity [26]. In addition, hippocampal volume seems to increase after an exercise intervention with high motor complexity (i.e., coordinative exercises) [27]. This finding is particularly important as increases in hippocampal volume are associated with improved brain function, while decreases in hippocampal volume are related to depression symptoms [27].

Taken together, it is reasonable to suggest that high motor complexity aerobic exercises may produce greater health benefits (depression score, cognitive function, hippocampal volume, BDNF expression, sleep parameters, cardiovascular risk parameters, suicidal behavior, functionality and treatment costs) than low motor complexity aerobic exercises (e.g., cycling, running). Thus, we hypothesized that aerobic exercise with motor complexity (AEMC) will be more effective than CAE in improving outcomes of cognitive decline, hippocampal volume, BDNF expression, sleep parameters, cardiovascular risk parameters, suicidal behavior, functionality, and treatment costs in patients with depression undergoing standard drug treatment.

## Methods/design

### Study design

**Fig. 1:**
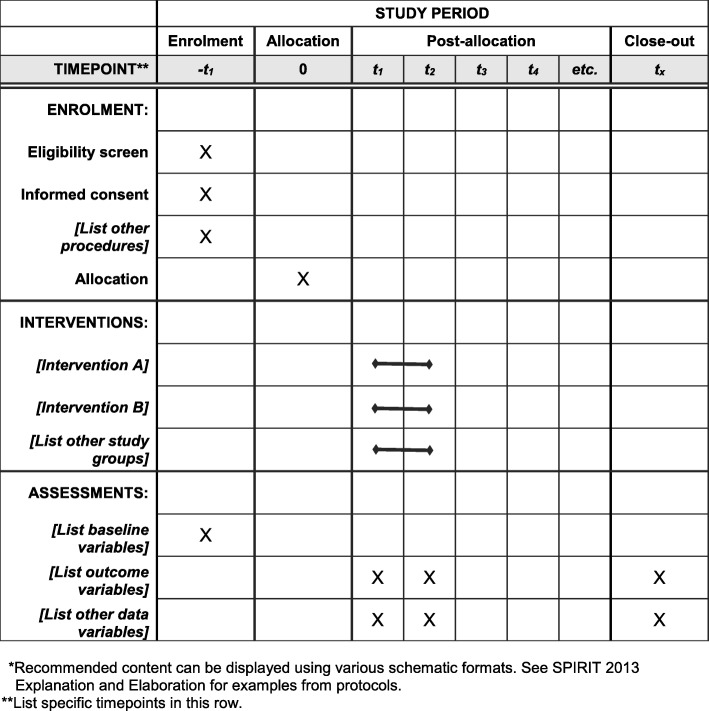
Template of recommended content for the schedule of enrolment, interventions, and assessments

This prospective, parallel group, single-blinded, superiority, randomized controlled trial will compare the effect of 24 weeks of either CAE or AEMC added to standard pharmacological treatment of patients with major depression in the Brazilian primary care system (Additional file 1 - SPIRIT 2013 Checklist) Fig. 1. Changes in depression score, cognitive function, hippocampal volume, BDNF expression, sleep parameters, cardiovascular risk parameters, suicidal behavior, functionality, and treatment costs will be assessed. It will be a single-blinded study since the researchers performing the assessments and statistical analyses will be blinded to treatment allocation. The expected start and completion dates are April 2018 and December 2018, respectively.

The primary outcome will be the change in the Montgomery-Åsberg Depression Scale (MADRS) score [28]. Secondary outcomes will be (1) cognitive function, assessed by Frontal Assessment Battery score [29], Trail Making Test A and B score [30], Rey’s Auditory-Verbal Learning Test (RAVLT) score [31], Digit Symbol Substitution Test score [32], Montreal Cognitive Assessment (MoCA) score [33], and Stroop test score [34]); (2) hippocampal volume assessed by magnetic resonance imaging (MRI) [35]; (3) BDNF expression [36]; (4) sleep parameters assessed by Pittsburgh Sleep Quality Index score [37], sleep quality assessment by accelerometry and the Biological Rhythms Interview of Assessment in Neuropsychiatry (BRIAN) score [38]; (5) cardiovascular risk assessed by global cardiovascular score, metabolic syndrome, waist circumference, blood pressure, lipid profile (total cholesterol, HDL cholesterol, LDL cholesterol, triglycerides), and glycemia [39, 40]; (6) suicidal ideation and behavior assessed by the Scale Columbia of Suicide score [41] and Scale of Hopelessness score [42]); (7) functionality assessed by gait linear spatial variables [43, 44], Timed Up and Go test (TUG) score [45], TUG cognitive score [46], handgrip strength [47], 6-min walk test [48] physical activity levels (sedentary time, active time, time spent in different physical activity intensities, energy expenditure, and number of steps per day), body weight, and hip and waist circumference [49]; and (8) treatment costs including laboratory procedures, medicines, and doctor consultations per patient [50].

The study will be conducted in accordance with theDeclaration of Helsinki revised in 2008 [51] and approved by the ethics committees of the Department of Health of the city of São Paulo and of the Hospital Israelita Albert Einstein, Certificate of Ethical Appreciation Presentation (PCEA) number 64888017.3.0000.0086. Trial registration is RBR-9zgxzd.

### Setting

**Fig. 2:**
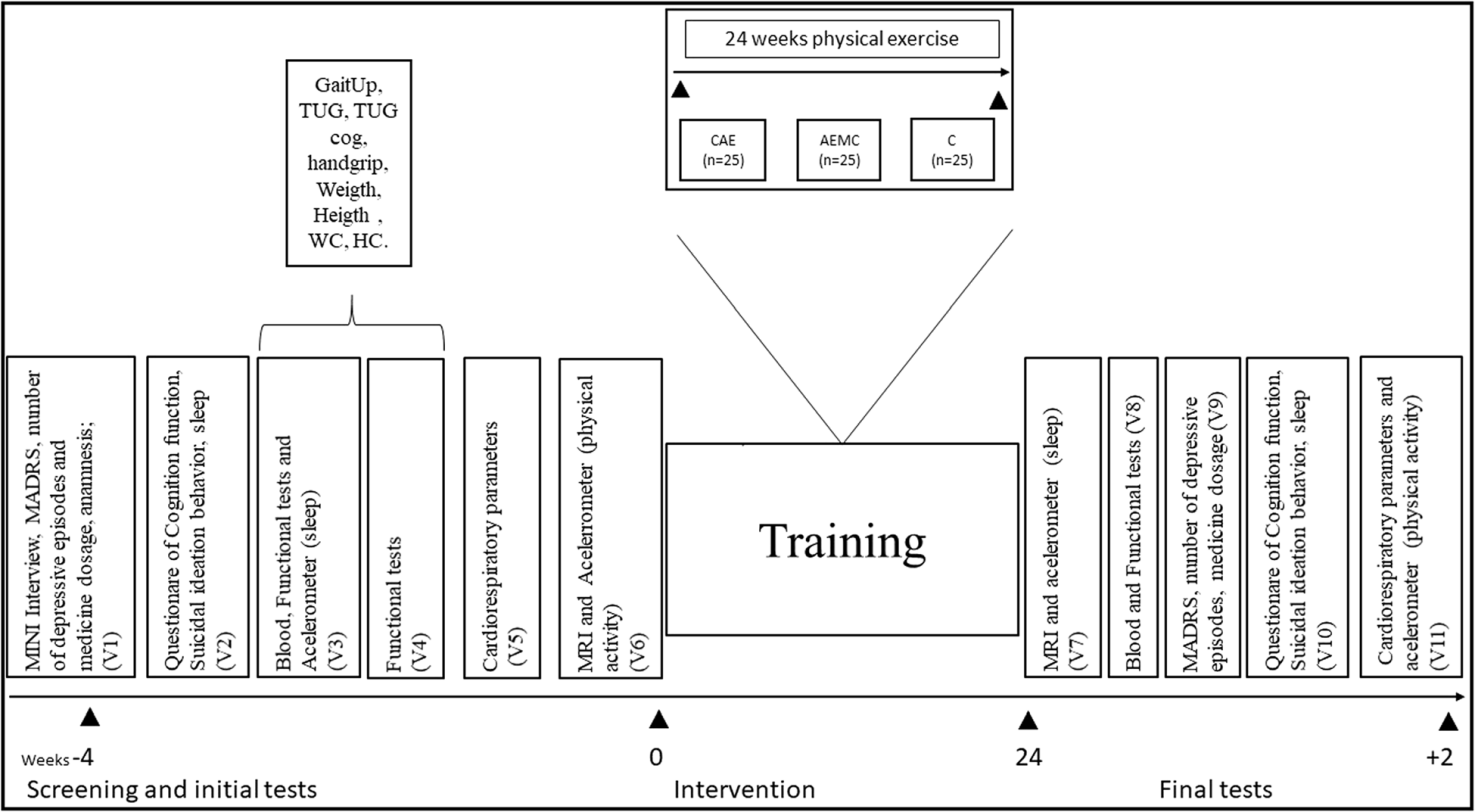
Study design. First 4 weeks, the recruitment and initial test. For 24 weeks, the intervention period – training. Final 2 weeks, the Final tests. Visit 1 (V1): Consent form, Mini International Neuropsychiatric Interview (MINI), MADRS, number of depressive episodes in life, medicine dosage in the last 3 months (necessary clinical adjustments), and anamnesis. Visit 2 (V2): Questionnaire of cognition function, suicidal ideation behavior and sleep; Visit 3 (V3): Blood sample, functional tests, and accelerometer (sleep); Visit 4 (V4): functional tests. Visit 5 (V5): cardiorespiratory parameters; Visit 6 (V6): MRI of the brain and sleep quality (physical activity). After 24 weeks of intervention, all of the experimental groups will be reassessed at visit 7 to visit 11 (V7 to V11). V7: MRI of the brain and accelerometer (sleep); V8: blood sample; V9: MADRS, number of depressive episodes and medicine dosage; V10: Questionnaire of Cognition function, suicidal ideation behavior, sleep; V11: Functional tests and accelerometer (physical activity). At the end of the study, patient treatment costs will be estimated consulting the medical records of each patient. *HC* hip circumference, *WC* waist circumference

Patients with depression will be recruited in the southern region of the São Paulo city at the Basic Health Unit (BHU; i.e., Arariba Park) in the state of São Paulo, Brazil. This BHU is part of the Brazilian Unified National Health System. First, patients will be referred by the physician team of the BHU if they fulfil the criteria of being 40 years and older, being treated for depression, and have no absolute contraindication to perform physical exercise. After the initial screening, patients will be fully informed of the procedures involved in the present trial and invited to sign an informed consent form. Participants will visit the laboratory on six occasions to perform the following assessments (Fig. 2):

- Visit 1: Consent form and anamnesis, Mini International Neuropsychiatric Interview (MINI) [52], and MADRS scale [28]. The number of depressive episodes in life and medicine dosage in the last 3 months (necessary clinical adjustments) [53] will be assessed by a trained psychiatrist researcher. Patients diagnosed with depression (single episode or recurrent episodes) will be referred to Visit 2. Patients will be informed that they may terminate their participation in this trial at any time, regardless of the reason.
- Visit 2: Questionnaire of cognitive function, suicidal ideation and behavior, hopelessness, and sleep quality.
- Visit 3: Blood samples after a 12 h fasting, functional tests, and instructions to use the accelerometer (GT9X ActiGraph accelerometer, Pensacola, USA) to assess sleep quality.
- Visit 4: these visits will be used to estimate the typical error of the functional tests (TUG test, TUG cognitive test, handgrip strength, 6-min walk test, body weight, hip and waist circumference); an interval of 48 h will be granted between Visits 3 and 4.
- Visit 5: Ergometric test and presence of absolute contraindications to exercise.
- Visit 6: MRI of the brain and instructions to use the accelerometer (GT9X ActiGraph accelerometer, Pensacola, USA) to assess physical activity level.

After 24 weeks of intervention, experimental groups will be reassessed in five visits (Visits 7–11; Fig. 2):

- Visit 7: MRI and accelerometer to assess sleep quality.
- Visit 8: Blood samples after 12 h of fasting.
- Visit 9: MADRS and necessary adjustments on medicine dosage [53] will be verified.
- Visit 10: Questionnaire of cognitive function, suicidal ideation and behavior, hopelessness, and sleep quality.
- Visit 11: Functional tests and accelerometer to assess the physical activity level.

At the end of the study, the patient treatment costs will be estimated [50, 54] by consulting the medical records of each patient.

### Type of participants

The sample will be composed of 75 patients with depression. Considering the heterogeneity of this population, the following inclusion criteria will be adopted: (1) diagnosed with major depression (single episode or recurrent episodes) as assessed by the MINI [55]; (2) pharmacological treatment for depression (use of antidepressants); (3) not engaged in regular exercise program (e.g., aerobic and/or strength training) for the 6 months prior to the commencement of experimental period; (4) absence of severe motor impairments, musculoskeletal injury, severe respiratory diseases, acute pulmonary embolism, unstable angina pectoris or severe heart failure, vascular brain disease, severe cardiopulmonary dysfunction, acute myocardial infarction or early phase of rehabilitation (critical aortic stenosis, severe hypertrophic and obstructive cardiomyopathy, untreated malignant arrhythmias, untreated severe hypertension, severe pulmonary hypertension), stroke or neurological disease, symptomatic cardiac malformations (e.g., septal defects, patent ductus arteriosus or valvular stenosis), atrioventricular block grade II or III, left bundle-branch block, or ventricular complex arrhythmias.

### Randomization

After baseline assessments, a blind researcher will classify patients into terciles regarding their MADRS score and number of previous depressive episodes. Subsequently, patients from each tercile will be randomly assigned to the control (C), CAE, or AEMC groups. A one-way ANOVA will test for similar initial values for MADRS scores and number of depressive episodes between groups (*P* > 0.05). The Principal Investigator will be blinded to all of the procedures to perform the randomization and analyses.

### Interventions

Both CAE and AEMC groups will exercise twice a week (on non-consecutive days) for 24 weeks at the Social Service of Commerce – Campo Limpo Unity. Each training session will last for approximately 60 min, with 10 min for warm up, 40 min dedicated to the actual exercise interventions, and 10 min for cool-down, following the American College Sports Medicine guidelines [56]. Training intensity will be maintained between 12 and 14 points of the rating of perceived exertion (RPE), which correspond to ~ 70 to 80% of the maximum heart rate [57]. All of the patients will be instructed to not engage in additional physical exercises throughout the intervention period. Individuals in the C group should not perform any exercise training, maintaining activities of daily living and regular visits to the BHU throughout the experimental period. The CAE group will perform continuous aerobic exercise. The AEMC group will perform aerobic exercises with progressive increases in motor complexity, involving displacements with movements of the limbs in phase, antiphase, and out of phase, usage of unstable bases of support and elements such as balls and discs, one-on-one tasks and changes in the external focus of attention (e.g., obstacles, paths, external rhythm). A room of approximately 10 m by 20 m will be used, where participants will perform a circuit (50 s of exercise and 30 s of rest) composed of 10 stations (each station corresponds to a different high motor complexity exercise). Exercise complexity on each station will be increased on an individual basis, as each patient learns how to perform the proposed motor activities. Exercise intensity will be determined by using the RPE scale. A heart rate monitor (H7 bluetooth, Polar, Kempele, Finland) using the app Polar Team (Polar Team, Polar, Kempele, Finland) will also be used to monitor exercise intensity. At the end of each training session, will also be obtained. The RPE scale is a widely used psycho-physical tool to assess subjective perception of effort during exercise [58]. The RPE scale is a 15-grade scale (6–20) in which exercise-related effort is classified in seven intensities (6–8 very, very light; 9–10 very light; 11–12 fairly light; 13–14 somewhat hard; 15–16 hard; 17–18 very hard; and 19–20 very, very hard) [59].

Together with the RPE, the two-sided scales of affective valence and perception of activation will be used [60]. The affective response will be assessed using the Feeling Scale (FS). FS is a single-item scale of 11 dimensions, ranging from − 5 to + 5, commonly used to measure affective valence (pleasure/displeasure) during exercise [60]. Patients will receive standardized instructions related to the use of FS prior to the exercise session. When explaining the FS, patients should understand that, while performing exercise, it is very common to feel a change in basic affect. Some people feel the exercise as pleasurable while others feel the exercise as unpleasant. Additionally, they will be informed that it is possible for a person to experience varying pleasure and displeasure during the exercise duration [48].

In all physical exercise sessions, four exercise scientists will prescribe and monitor the interventions. Exercise scientists have already completed a 20 h workshop to standardize exercise prescription protocols.

### Mini International Neuropsychiatric Interview (MINI)

The Portuguese version of the 7.0.2 MINI will be used to screen patients for symptoms of major depression. The MINI has a sensitivity and specificity of 95% and 84%, respectively [52]. Questions are phrased to allow only ‘yes’ or ‘no’ answers. The MINI explores all the inclusion and exclusion criteria and chronology of 23 diagnostic categories of the DSM-V and has demonstrated satisfactory reliability [61] for all diagnostic sections (except for the section on psychotic disorders). Specifically for major depression, negative responses in questions A1 (A and B) and A2 (A and B) exclude the possibility of the disease. In the case of affirmative answers to questions A1 or A2, seven additional questions are asked (A3; (A) appetite; (B) trouble sleeping; (C) move more slowly than normal; (D) feel tired; (E) feel worthless or guilty; (F) difficulty concentrating; (G) repeatedly think about death). If five questions are positive (in A1–A3), the patient is diagnosed with major depression. Two more questions are asked (A4 and A5) to check if it is a recurrent episode.

### Outcome measures – primary outcome

The MADRS scale [28] encompasses 10 items that assess apparent and reported sadness, inner tension, sleep and appetite changes, concentration difficulties, slowness, inability to feel, and pessimistic and suicidal thoughts. Nine items are based on the patient’s report and one on the psychiatrist’s observations. The higher the score, the greater the presence of depressive symptoms; scores range from 0 to 60 [62]. The MADRS scale will be applied in the pre- and post-intervention assessments.

### Outcome measures – secondary outcomes

#### Clinical outcomes – cognitive function

The Frontal Assessment Battery [29] is a tool designed to evaluate executive function. This instrument takes approximately 10 min to administer its six items, namely (1) similarities (conceptualization) – explores abstract reasoning by presenting pairs of objects from the same semantic category; (2) lexical fluency (mental flexibility) – assesses self-organization, strategy, and change, generating as many words as possible that start with a given letter; (3) motor series – explores motor programming and planning by carrying out Luria’s series (fist-side palm); (4) conflicting instructions – explores sensitivity to interference, giving the opposite response to that of the examiner; (5) go/no-go – assesses inhibitory control and impulsivity; and (6) prehension behavior – assesses the ability to spontaneously inhibit prehension. A maximum score of 18 is obtained adding the score on each of the six items, which are scored between 0 and 3. Each of the six items assesses cognitive and behavioral domains involved in different neural networks.

The Trail Making Test (sections A and B) was developed in the middle of the last century [30] to evaluate visual tracking, attention, mental flexibility, and motor function [63]; it has been used to evaluate the effect of physical exercise on executive functions [20]. The test is divided into two sections; section A requires the connection in ascending order of 25 numbers within circles arranged randomly on an A4 sheet and section B requires the connection between 12 letters and 13 numbers alternately in alphabetical and ascending order [64]. The test should be performed as fast as possible without lifting the pen or pencil from the paper. The score is obtained as the time (seconds) spent to complete sections A and B. The higher the value on these two sections, the worse the participant’s performance.

The RAVLT was originally developed by Andre Rey [31] and became one of the most used memory assessment tests; it was translated, adapted, and standardized to Portuguese. It consists of two lists of 15 nouns (A and B) and a list of distracting words. List A is read five consecutive times (A1, A2, A3, A4, and A5). Immediately after each reading, the patient must repeat the recollected words. Subsequently, list B is read, and the patient has to repeat the recollected words (B1). After reading list B, the patient should verbalize the remembered words from list A (A6). After a 15-min interval, the patient must identify the words from list A from a list of 30 words (A7).

The Digit Symbol Substitution test [32] consists of filling in as many empty boxes as possible using a symbol that corresponds to a number. Patients have up to 90 s to complete the task. The strategy to solve the Digit Symbol Substitution test requires speed of response, sustained attention, visuospatial skills, and cognitive alternation [65]. The top of the form used to apply the test presents nine symbols with corresponding numbers (1 to 9). The subject must write the numbers corresponding to each symbol each time it is presented (up to 120 situations). Performance is measured by the number of correct answers in a 90 s period, with the score ranging from 0 (worst performance) to 120 points (best performance).

The MoCA [33] was designed to assess cognitive impairment (0 to 30 points) in seven cognitive domains, namely visuospatial and executive function (5 points), appointment (3 points), attention (6 points), language (3 points), abstraction (2 points), late evocation (5 points), and orientation (6 points). Recent evidence suggests that MoCA can also be used as an outcome to determine changes in cognitive function after physical exercise interventions in different populations [66–69].

The Stroop test [34] uses three cards containing 24 stimuli each and a white background. Card A is composed of rectangles printed in green, pink, blue, and brown, randomly arranged. Card B is organized similarly to card A, but with rectangles replaced by the words ‘every’, ‘never’, ‘today’, and ‘all’ printed in capital letters in the four colors mentioned. Card C was also organized similarly to card A, representing the interference card in which the name of the colors (brown, blue, pink, and green) printed and written on each card never matches (e.g., brown word printed in pink, green, or blue). For the first card, participants have to state the colors of the rectangles as quickly as possible. For cards B and C, subjects must state the color of the printed words and not the written words. The criterion score was the time taken to perform the task relative to each card.

#### Hippocampal volume

All scans will be performed using a 3 T Magnetom Verio MRI scanner (Siemens Medical Systems, Erlangen, Germany) using a single channel head coil. High-resolution anatomical images will be recorded in the sagittal plane using a three-dimensional inversion recovery gradient echo sequence (Sequence MPRAGE, gradient mode FAST, magnetic preparation NON-SEL, IR, RF pulse type FAST, excitation NON SEL, TR = 2300 ms, TE = minimum, TI = 900 ms, flip angle 9SEG, bandwidth 240H/PIX, base resolution 192, averages 1, concatenations 1, phase oversampling 0%, FOV phase 100%, FOV read 240–256 mm^2^, slice thickness 1.2 mm, slice per slab (number of slices) 160–170, Filter NONE, parallel acquisition technique (iPAT) OFF, Prescan Normalize ON).

The image processing for volumetric determination will use the NeroQuant system^®^ [35] (CorTechs Labs, Inc., San Diego, CA, USA). CorTechs Labs is an FDA 510(k) cleared and CE marked medical device software that automates the quantification of segmented brain structures from 3D T1 MR images. The resulting brain structure volumes are compared to a healthy population of age- and sex-matched normative reference data, which provides clinicians with a framework to better understand brain structure volumes when assessing patients. Output includes disease-specific volumetric data and color-coded images of brain structures. The 3D T1 MR images are uploaded to the NeuroQuant^®^ software for processing. The images are registered to patient brain space. Brain structures are identified and labeled using CorTechs Labs’ Dynamic Atlas technology. The reports and segmented images are then sent back to the researcher via the PACS system.

#### BDNF level

Blood samples will be used to determine BDNF concentration according to the World Health Organization Guidelines [40] after a 12 h overnight fast. The methodological procedures regarding storage, manipulation, and analysis of blood samples will follow the recommendations of Pareja-Galeano et al. [36]. Blood samples will be collected in tubes without additives and coagulated for 24 h at 4 °C. Samples will then be centrifuged at 1500 rpm for 15 min at 4 °C and the supernatants will be frozen at − 20 °C. The levels of BDNF in all samples will be measured using ELISA kits (Mature BDNF RapidTM ELISA Kit Human, BEK-2211-1P/2P, Biosensis, Australia).

#### Ergometric test

The ergometric test will be performed by a physician with extensive experience. Patients will be instructed to not perform exhaustive physical activities and ingest caffeinated and alcoholic beverages within 48 h prior to the test. Tests will be performed in a controlled temperature environment (21–23 °C). Resting electrocardiogram will be performed with 12-lead monitoring, with a previous 5 min rest period (APEX 200, TEB^®^, São Paulo, Brazil) will be used. An incremental test will be performed up to the tolerance limit using a treadmill (APEX 200). Throughout the test, the electrocardiographic signal will be recorded (APEX 200). The exercise protocol will start at 3.1 km h^− 1^, with progressive increases of the slope by 1 or 1.5% every 60 s, until voluntary exhaustion. The criteria for test interruption will be in accordance with the Guidelines of the Department of Ergonomics of the Brazilian Society of Cardiology [70].

#### Cardiovascular risk

##### Overall cardiovascular score

The Brazilian Cardiovascular Prevention Directive recommends the use of the Global Cardiovascular Score [71] as an indicator of cardiovascular risk. For its calculation, age (ratings of − 9 to 8), total cholesterol (ratings of − 3 to 3), HDL-cholesterol (ratings of − 3 to 5), systolic blood pressure (ratings of − 3 to 3), a diagnosis of diabetes (ratings of 0 to 4), and whether the individual is a smoker (ratings of 0 to 2) are considered. After attributing the values of the respective outcomes, the sum of these results in a single value, which corresponds to cardiovascular risk. This ranges from less than 0 (no risk) to greater than 17 (cardiovascular event risk of 30%). Health professionals receive a booklet with details of such calculation from the Brazilian Ministry of Health [72].

#### Metabolic syndrome

The metabolic syndrome is characterized by the presence of three or more conditions from changes in arterial pressure (systolic pressure ≥ 130 mmHg or diastolic pressure ≥ 85 mmHg), fasting blood glucose (≥ 110 mg/dL), central obesity (abdominal circumference, men > 102 cm and women > 88 cm), dyslipidemia (high LDL-cholesterol, men > 40 mg/dL and women > 50 mg/dL), and triglycerides (> 150 mg/dL) [73].

#### Blood pressure and heart rate variability

Blood pressure and heart rate variability will be obtained in the supine position after 10 min of rest by an evaluator blinded to other assessments and group allocation. Brachial auscultatory blood pressure will be measured in triplicate on both arms using a sphygmomanometer and a stethoscope (BIC, Standard, Brazil) considering phases I and V of the Korotkoff sounds for the identification of systolic and diastolic blood pressures, respectively. The mean of three measured values, with at least 4 mmHg of difference between them on each arm, will be calculated. The mean value obtained on the arm with the higher blood pressure will be considered for the analysis [74].

For the determination of resting heart rate variability, RR intervals will be obtained by a heart rate monitor (POLAR – V800, USA) for 10 min. The evaluator will visually inspect all RR intervals and correct, via interpolation, any inappropriate or premature RR interval. Any RR interval with a difference greater than 20% of adjacent intervals will be filtered using a moving average algorithm. Normalized RR intervals will be transferred to a dedicated heart rate variability software package (Kubios HRV v 2.1, University of Eastern Finland, Finland) for further analysis. Using the recommendations of the Task Force for heart rate variability, the time-domain (SDNN, RMSSD, and pNN50) and frequency-domain parameters (low frequency, high frequency, and ratio between low and high frequency) will be analyzed and a non-linear analysis (SD1 and SD2) will be performed as previously described [75].

#### Blood sample collection

All fasting blood samples will be collected in additive-free tubes (serum), in K3-EDTA containing tubes (plasma), and in clot activation gel tubes (whole blood). Whole blood will be frozen immediately after collection of blood samples and stored at − 20 °C. The serum and plasma samples will be centrifuged at 3000 rpm for 15 min at 4 °C. Supernatants will be frozen and stored at − 20 °C until analysis. These samples will be analyzed for lipid profile (total cholesterol, HDL, LDL, triglycerides) and blood glucose levels (Ultrospec 6300 Pro), according to World Health Organization Guidelines [40] and the manufacturer’s instructions.

#### Clinical outcomes – scales of suicidal ideation, sleep, and BRIAN

The Columbia Suicide Severity rating scale distinguishes suicidal ideation from suicidal behavior. There are four subscales, namely severity of ideation (score of 0–5), intensity of suicidal ideation (score of 0–5), behavior (which is a nominal scale that evaluates actual, aborted, interrupted attempts, and self-injurious behavior, with no intent to commit suicide), and the fourth is the lethality of the actual attempts (scale score 0–6) [41].

The Scale of Hopelessness identifies the level of pessimism, which is directly related to the chance of developing several mental disorders and to suicide risk. The scale consists of 20 true–false questions. The total score is 20 points and each item is scored as 0 or 1 [42].

The Pittsburgh Sleep Quality Index score is a tool widely used for research and practice regarding psychiatric disorders [37]. This questionnaire interprets the perception of sleep quality in a feasible and reliable manner [37, 76]. The overall index is composed of seven components, namely subjective sleep quality (perception of sleep quality of subjects), sleep latency (time between lying on bed and sleeping), duration of sleep (sleeping period reported by the individual), sleep disturbance (sleeping problems), sleep medication (use of sleeping medications), and diurnal dysfunction (daytime sleepiness intensity). All the seven components are scored from 0 (no difficulty for the component of interest) to 3 (very bad). The scores on these components are combined to generate a global score that reflects the sleep quality and disturbances over a 1-month period. The overall score ranges from 0 to 21; if a patient reaches at least 5 points, they are diagnosed with an alteration of sleep. The higher the score the worse the quality of sleep.

The BRIAN is a self-report questionnaire designed to provide an index of disturbances in the circadian rhythm [38]. This scale considers sleep interruptions, irregularities in social rhythm, eating patterns, and abnormalities in daily activity at home and at work in the last 15 days, all of which are postulated to contribute to the onset and worsening of affective episodes, as well as psychosocial function and clinical outcomes [77–80]. The questionnaire includes 18 items divided into four domains of the circadian biological rhythm, namely sleep (5 questions), activity (5 questions), social (4 questions), and food patterns (4 questions). Each question is scored in 4-point subscales, with scores ranging from 1 (no difficulties in maintaining the usual pace) to 4 (serious difficulties in maintaining the usual pace), totaling a minimum and maximum overall score of 18 and 72, respectively. Higher scores indicate a greater rupture of the circadian rhythm. The chronotype is also captured by BRIAN, as the last three items address the individuals’ daily preference for work and sociability, if they feel more productive in the morning, and if they feel that their day and night cycle has been reversed over the last year. Three questions about circadian preference evaluate the morning and evening chronotypes: “Do you have more energy for work and interpersonal relationships at night?” (Active at night), “Do you feel more productive in the morning?” (Active in the morning), and “Have you reversed your day/night cycle?” (Reverse day/night cycle). The options are never (score of 1), rarely (score of 2), often (score of 3), or always (score of 4). A low score indicates a morning preference and a high score indicates a night preference [81].

##### Clinical outcomes – functional tests

Linear spatial variables of gait will be assessed using three inertial sensors (GaitUp, Physilogs^®^, Switzerland), while participants walk on a 15 m walkway. Each of the three sensors encompasses a micro-controller, memory, three-axis accelerometer (range ± 3 g), a gyroscope (range ± 800° s − 1), and a battery comprising a small (50 mm × 37 mm × 9.2 mm) and lightweight module (19 g). Two modules are fixed in the foot and one is fixed in the lower back. These sensors will measure step length, gait speed, cadence, and percentage of oscillation, variables that have been previously validated to assess gait pattern in older adults [43, 44]. The participants will perform three trials on three different conditions, namely (1) single task (only walking); (2) dual task words (walking and speaking words); and (3) dual task subtractions (walking and serial –3 subtractions).

The TUG test [45] is one of the most used tests to assess the functionality of older individuals. The patient will be timed while rising from a chair (seat height 46 cm), walking as quickly as possible at a comfortable and safe pace to a line on the floor 3 m away from the chair, turning and walking back to the chair, and siting down again. Time will be recorded from the instant the patient’s buttocks leave the chair (standing up) until the next contact with the chair (sitting down). Before the test, the patients will perform two familiarization attempts separated by 1 min. After the familiarization trials, two test trials will be performed with a 1 min interval between trials [37]. The lowest time will be used for analysis.

The TUG cognitive test [46] will be identical to the TUG, but individuals will perform cognitive tasks (subtractions) during the test. The patients will say the results of serial –3 subtractions starting with different numbers [46] to eliminate any training effect. The lowest time will be used for analysis.

The handgrip strength will be assessed using a Jamar dynamometer (Lafayette Instrument Co, Lafayette, USA). The instrument has two parallel handles, one fixed and one movable. This instrument has a closed hydraulic system that measures the amount of grip strength when producing an isometric contraction of the hand muscles. Results will be recorded in kilograms. Patient’s position will follow the guidelines of the American Society of Hand Therapists [47], which positions the shoulder slightly adducted, the elbow flexed at 90°, and the forearm in a neutral position. The testing protocol consisted of three 5 s maximal isometric contractions performed with the dominant hand, with a rest period of at least 60 s. Two attempts will be performed on 2 separate days (test and retest, minimum interval of 48 h). The highest force value between the 2 days will be considered for analysis.

The 6-min walk test is an inexpensive, relatively fast, safe, and well tolerated method for assessing exercise capacity [48]. It consists of walking as far as possible in 6 min in a 30-m track [48]. Previous studies demonstrated the agreement (*r* = 0.59) (standard error of estimate = 3.82 mL/kg/min) of this indirect protocol with the gold standard protocol for VO_2max_ [82].

#### Physical activity level and sleep parameters

Physical activity level and sleep parameters will be assessed by a tri-axial accelerometer (GT9X Actigraph, Pensacola, USA). For the physical activity assessment, patients will wear the accelerometer [83] for at least 7 days, with a sampling rate of 100 Hz. The patient will be instructed to not change their routine and to wear it from the moment of waking to sleeping time, removing it just to shower. The equipment will be worn on the waist (right side) as directed by the manufacturer. The observed parameters for physical activity will be sedentary time, active time, time spent on different physical activity intensities, energy expenditure, and number of steps. For the sleep parameters, patients will be instructed to not change their routine and to remove the accelerometer just to shower (sleep with accelerometer). The equipment will be worn on the wrist (non-dominant side) as directed by the manufacturer. The observed parameters for sleep will be (1) total sleep time (i.e., total sleep time, not including awaking time in bed), calculated as the total number of sleeping minutes from ‘lights off’ to ‘lights on’; (2) sleep onset latency (i.e., amount of time spent in the transition from wakefulness to sleep in minutes), a higher sleep onset latency indicates disturbed sleep (difficulty falling asleep); (3) sleep efficiency (i.e., ratio of total sleep time to bed time expressed as a percentage), a lower sleep efficiency indicates a lower sleep quality; (4) wake after sleep onset (i.e., sum of all moments (minutes) between onset of sleep and end of sleep – early onset); wakefulness after sleep onset will be recorded in minutes, a longer awake period after sleep onset indicates a more fragmented sleep; and (5) total activity count (i.e., sum of activity counts every night during sleep, on average, during the 7 days of use of ActiGraph) [84]. Regarding the use of the accelerometer to assess habitual physical activity and sleep, patients will receive a copy of the user guide, highlighting the importance of wearing it for 7 days, as well as the correct moment and position of use.

#### Clinical outcomes – Body composition

Body weight will be assessed using an electronic scale (W200, Welmy, Brazil). Height will be measured using a fixed stadiometer (Sanny, São Paulo, Brazil) with an accuracy of 0.1 cm. Weight and height values will be used to calculate the BMI. Measurements of hip and waist circumference will be obtained with the use of an anthropometric metal tape with precision of 0.1 cm. All anthropometric measures will be performed following the standardization described by Guerra et al. [49].

#### Treatment costs

Treatment cost for each patient will be determined using the methods described by Codogno [50, 54]. At the end of the trial, the demands for services and medicines recorded on medical records throughout the 6 months of the intervention period will be considered. The following information will be obtained: medicines supplied to the patient, laboratory tests performed, and the number of consultations throughout the intervention period. In addition, patient costs will encompass the cost of administrative and maintenance services related to the BHU where the procedures will be performed. The costs stipulated for these services and the number of patients attended will be added to the costs of each patient, according to the number of days that they visited the BHU over the 6-month period. In order to transform the procedures into actual currency, the values informed by the Municipal Health Department for the year 2018 will be used.

### Data analysis and power calculations

The Shapiro–Wilk and Levene’s tests will be used to determine normality and variance equality, respectively. Non-normal data will be log transformed. If the transformation does not produce a normal distribution, the effect of influential points in data analyses will be tested. If influential points change the interpretation of the results, they will be manually removed. Linear mixed models having group and time as fixed factors and patients as a random factor will be performed for each outcome (MADRS, clinical outcomes, hippocampal volume, brain-derived neurotrophic factor, cardiopulmonary parameters, lipid profile, and treatment costs) and domain (especially in clinical outcomes) [85]. In case an outcome presents pre-experimental period differences between groups, determined by a regular one-way ANOVA, an ANCOVA will be implemented having pre-experimental period values as covariates, group as a fixed factor, and patients as a random factor. An intention-to-treat analysis will also be performed in case of a high incidence of missing values, using linear mixed models; the first analysis will be performed removing the missing values, while the second analysis will be performed incorporating the missing data points to determine the impact of missing values in the overall results. Whenever a significant F-value is obtained, a post hoc test with a Tukey’s adjustment will be performed. In addition, within-group (pre- to post-changes) and between-group effect sizes will be calculated using Cohen’s d [86] for each outcome. Effect sizes will be classified as small (≤ 0.49), medium (0.50–0.79), and large (≥ 0.80). Finally, the level of significance adopted in the present study for all analyses will be *P* ≤ 0.05. Data will be presented as mean ± SD. SAS 9.2^®^ software (Institute Inc., Cary, NC, USA) will be used for the statistical analyses described. Considering the importance of the control of the error rates, typical error and coefficient of variation of the assessments will be performed under test and retest conditions in a sub-sample (10 subjects).

For the primary outcome (MADRS) the verification of status of depression (depressed, partial remission, full remission) and the number of patients in remission (< 10 points MADRS) will be verified [14, 87].

Power calculations were performed using data published by Kerling et al. [88] (effect size 0.25), who showed that an aerobic exercise program can positively change depression scores as assessed by the MADRS scale. The obtained sample size was 21 patients per group (power 0.85, alfa error 0.05, correlation between repeated measures of 0.8). Assuming a drop-out ratio of approximately 30%, we will determine 25 patients per group as appropriate.

## Discussion

The present study has the objective to verify if patients with depression randomized into one of two physical exercise interventions (AEMC or CAE) combined with traditional drug treatment will present distinct improvements in depression symptoms (MADRS). In addition, different outcomes commonly altered by depression and with the potential to be reversed by physical exercise (e.g., cognitive function, hippocampal volume, brain-derived neurotrophic factor, sleep parameters, cardiovascular risk parameters, suicidal behavior, functionality, and treatment costs) will be evaluated.

From an operational standpoint, a pilot study involving 45 healthy elderly people was carried out in 2017, considering interventions with AEMC (two levels) and CAE (stationary cycling). Data from the pilot study are under analysis, but greater improvements with regards to cognitive and motor aspects were observed in the group undergoing exercises with greater motor complexity. In addition, training interventions produced very high exercise adherence (95%), which may be a differential, considering the difficulties in maintaining adherence during a traditional physical exercise program [89]. The main objective of this pilot study was to establish training routines and assessments and to provide logistical experiences between the parties involved (BHU, University, institutions involved in implementing training interventions and evaluations, and researchers) for the development of the proposal with patients with depression.

To our knowledge, this is the first study to test the effect of adding physical exercise to traditional treatment in patients with depression in a primary health care setting in Brazil. In addition, the use of aerobic exercises with insertion of activities with progressively higher motor complexity has not yet been tested in this population. Considering that exercises with high motor complexity may promote changes in cortical activation [90] and possibly increase the volume of associated encephalic structures (i.e., hippocampus) [27] in healthy individuals, assessing such adaptations in patients with depression is of great interest for clinical practice and public policies.

Finally, determining the impact of physical exercise programs on the treatment costs of patients with depression in Brazil may lead to new polices on how to treat them while decreasing the associated costs [2].

This study was selected from the Research program for the Brazilian Unified National Health System – “PPSUS 2015” [91], and was financed by the following Brazilian public institutions: São Paulo Research Foundation (FAPESP), State Government of São Paulo, Brazilian Ministry of Health, National Research Council (CNPQ) and Coordination for the Improvement of Higher Education Personnel.

### Trial status

No participants have been included in the study. Data collection will start on April 2018 and is expected to be concluded on December 2018. This is version 2 of this study. Version 1 was submitted to FAPESP (Funding agency) on July 29, 2015. The changes in version 2 were the use of MINI as a screening toll for depression, MADRS as the primary outcome for depression, inclusion of MRI for brain structure assessment, the use of a questionnaire for suicidal ideation and behavior, sleep parameters, and sleep-related accelerometry data.

### Ethics and dissemination

This trial is approved by the ethics committee of the Hospital Israelita Albert Einstein and the Department of Health of the city of São Paulo (CAAE) number 64888017.3.0000.0086. Trial registration is RBR-9zgxzd. According to Brazilian legislation, individual data will be stored for 5 years, and patient identification will not be made publicly available. As approved by the ethics committee, patients in need for ancillary care will be referred to the Brazilian public emergency service. As provided by the financing notice, at the end of the study the Principal Investigator should produce a research report with a focus on its use in the management of the Brazilian Unified National Health System.

## Additional file

Additional file 1: SPIRIT 2013 Checklist: Recommended items to address in a clinical trial protocol and related documents*. (PDF 116 kb)

## Abbreviations

AEMC: Aerobic exercise with motor complexity
BDNF: Brain-derived neurotrophic factor
BHU: Basic Health Unit
BRIAN: Biological Rhythms Interview of Assessment in Neuropsychiatry score
C: Control
CAE: Continuous aerobic exercise
FS: Feeling Scale
MADRS: Montgomery-Åsberg Depression Scale
MDD: Major depressive disorder
MINI: Mini International Neuropsychiatric Interview
MoCA: Montreal Cognitive Assessment
MRI: Magnetic resonance imaging
RAVLT: Rey’s Auditory-Verbal Learning Test
RPE: Rating of perceived exertion
TUG: Timed Up and Go test
VO_2peak_: Peak oxygen uptake
